# Genome-Wide Identification of the *DFR* Gene Family in *Lonicera japonica* Thunb. and Response to Drought and Salt Stress

**DOI:** 10.3390/genes16121453

**Published:** 2025-12-04

**Authors:** Dandan Lu, Xiaoyu Su, Yao Sun, Lei Li, Yongliang Yu, Chunming Li, Yiwen Cao, Lina Wang, Meiyu Qiao, Hongqi Yang, Mengfan Su, Zhengwei Tan, Huizhen Liang

**Affiliations:** 1 Institute of Chinese Herbal Medicines, Henan Academy of Agricultural Sciences, Zhengzhou 450002, China; ludandan0710@163.com (D.L.); suxiaoyu_2014@163.com (X.S.); ruth_3912834@163.com (Y.S.); 15136189572@163.com (L.L.); yyl790721@126.com (Y.Y.); lchm1212@163.com (C.L.); yiwencao96@163.com (Y.C.); hnndlina@163.com (L.W.); qiaomeiyu123@163.com (M.Q.); yang13303833929@163.com (H.Y.); sufancy1669@163.com (M.S.); 2 Provincial Key Laboratory of Conservation and Utilization of Traditional Chinese Medicine Resources, Zhengzhou 450002, China

**Keywords:** *Lonicera japonica*, DFR, abiotic stress, expression pattern, gene cloning

## Abstract

**Background:** Dihydroflavonol 4-reductase (DFR) is pivotal for anthocyanin biosynthesis and plays a crucial role in plant development and stress adaptation. However, a systematic characterization of the *DFR* gene family is lacking in *Lonicera japonica* Thunb. **Methods:** In the present study, based on genome and transcriptome data of *L. japonica*, the research identified six *LjDFR* gene family members throughout the entire genome. **Results:** The *LjDFR* genes were located on Chr.04 and Chr.09 and the full-length coding sequences of *LjDFR1*-*LjDFR6* were cloned. Subcellular localization analysis showed that LjDFRs are primarily found at the cell membrane and in the nucleus. Phylogenetic analysis showed closer clustering of *LjDFR* genes with *Capsicum annuum* and *Camellia sinensis*. Promoter analysis linked *LjDFR* genes to light response, hormone signaling, and stress-responses. qRT-PCR analysis demonstrated tissue-specific and stage-specific expression patterns among *LjDFR* members. Notably, *LjDFR2* expression was significantly higher in the intensely pigmented tissues of *Lonicera japonica* Thunb. var. *chinensis* (Wats.) Bak. compared to *L*. *japonica*. Coupled with its phylogenetic proximity to the anthocyanin-related *CsDFRa* and *CaDFR5* genes, this suggests that *LjDFR2* may be positively correlated with anthocyanin accumulation. Additionally, the expression of *LjDFR2* and *LjDFR4* was markedly induced by both drought and salt stress, indicating their roles in abiotic stress responses. **Conclusions:** This research provides a foundation for further functional studies of *LjDFR* genes in anthocyanin biosynthesis and stress resistance and offers candidate genes for molecular breeding of *L*. *japonica*.

## 1. Introduction

Anthocyanins are water-soluble pigments found in plants. They belong to a group of flavonoid-derived compounds and are mainly stored in the vacuoles of flowers, fruits, seeds, and leaves [1]. In plants, anthocyanins perform several important things, including attracting pollinators, repelling herbivores, activating chemical defenses, and protecting against pathogens, UV rays, low temperatures, and drought [2]. Due to their strong antioxidants, anthocyanins also offer health benefits for animals and humans, such as controlling blood sugar, lowering blood fats, and may reduce cancer risks [3]. About 600 types of anthocyanin exist, with six main types: delphinidin, pelargonidin, petunidin, cyanidin, peonidin, and malvidin; cyanidin is the most common. In terms of color, cyanidin and pelargonidin provide red hues, while delphinidin and its derivatives like petunidin and malvidin give blue-purple shades [4]. The final color of anthocyanins is influenced by factors like co-pigmentation, vacuolar pH, structural differences, and metal ions [5].

The anthocyanin biosynthetic pathway is part of the flavonoid biosynthesis. It is the end product of the phenylpropanoid/flavonoid pathway and involves several enzymes [6]; DFR is the rate-limiting enzyme. It directs carbon flow, leading to different types of anthocyanins [7]. DFR can transform three colorless dihydroflavonols (dihydrokaempferol DHK, dihydroquercetin DHQ, dihydromyricetin DHM) into leucoanthocyanidins. Thus, DFR is a key regulator in anthocyanin production [8]. There are three types of DFRs: the first is the Asn-type DFRs, found in many plants, which can process all three dihydroflavonols; the second are the Asp-type DFRs, which are specific to DHQ and DHM, and cannot effectively catalyze DHK; the third type are called non-Asn/Asp-type DFRs, containing neither Asn nor Asp [9]. Biochemical studies show that DFR proteins have specific substrates, which affect the types and amounts of anthocyanins, leading to color variations [10]. For instance, petunia DFR cannot process DHK, so this species lacks orange flowers. However, when certain amino acids in the DFR enzyme are changed, the modified petunia DFR can process DHM, resulting in orange flowers with pelargonidin-based anthocyanins [11]. Therefore, DFR is crucial in the anthocyanin pathway, and adjusting its expression can change plant color.

**Figure 1:**
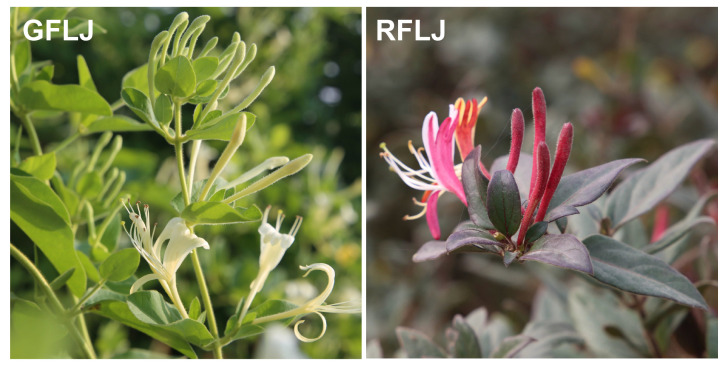
The two *L. japonica* lines.

*L. japonica* (Caprifoliaceae), commonly known as Jin-Yin-Hua or Flos Lonicerae Japonicae (FLJ), refers to the dried flower buds or nearly open flowers of the plant. It is perennial semi-evergreen climbing shrub blooms from April to June; its flowers transition in color from pure white to golden yellow, often displaying both hues simultaneously on the same vine, hence the name “Jin-Yin-Hua” (Figure 1). This plant is highly adaptable, tolerant to cold, heat, and drought, but susceptible to waterlogging. As a traditional Chinese herb, *L*. *japonica* possesses various biological activities, including antibacterial, anti-inflammatory, antiviral, antioxidant, anti-endotoxin, hypolipidemic, and antipyretic effects [12]. It is also an effective antiviral against SARS coronavirus, influenza A virus, and the novel coronavirus [13]. *Lonicera japonica* Thunb. var. *chinensis* (Wats.) Bak. (RFLJ) is a natural variant of *L*. *japonica*. Its young branches, leaves, and stems are all purplish-red, with a purplish-red corolla outside and white inside (Figure 1). Compared to *L*. *japonica*, RFLJ has more luteoloside, quercetin, chlorogenic acid, and anthocyanins, along with a wider range of volatile oils. Research by Yuan et al. shows that RFLJ can have anthocyanin levels as high as 100 mg/100 g [14]. *L*. *japonica* flowers are colorful and fragrant, passing through six stages: S1 (young alabastrum), S2 (green alabastrum), S3 (slightly white alabastrum), S4 (whole white alabastrum), S5 (silvery flower), and S6 (golden flower). The differences in anthocyanin levels and flower color at S4 separate *L*. *japonica* (GFLJ with green flowers) from RFLJ (with purple flowers) [15].

Currently, research on DFR mainly focuses on gene cloning and functional identification. The *DFR* gene family has been identified in only a few species, such as *C. annuum* [16] and *C. sinensis* [17], while a comprehensive identification or systematic study of the *DFR* gene family in *L*. *japonica* has been lacking. To address this knowledge gap and investigate the role of *LjDFR* in the regulation of anthocyanin biosynthesis and its function in stress response, we conduct a comprehensive analysis of the *LjDFR* genes, including physicochemical properties, chromosomal localization, collinearity, conserved protein domains, gene structures, and promoter cis-acting elements, subcellular localization, tissue-specific expression and expression levels under drought and salt stress. This research will lay a foundation for further exploring the function of *LjDFR* genes in the anthocyanin metabolic pathway and provides theoretical guidance for the breeding of new *L*. *japonica* varieties.

## 2. Materials and Methods

### 2.1. Materials and Stress Treatments

The test materials, GFLJ and RFLJ (Figure 1), were collected from Fengqiu, Henan, and maintained by the Institute of Chinese Herbal Medicines, Henan Academy of Agricultural Sciences. They were grown under natural conditions at the Modern Agricultural Research and Development Base of Henan Academy of Agricultural Sciences (34°55′~35°11′ N, 113°36′~114°15′ E). During the first flowering stage, roots, stems, leaves, and flower buds of 5–10 plants were collected from both varieties, along with flowers at various stages. Each sample had three biological replicates. After quickly freezing in liquid nitrogen, samples were stored at −80 °C for later use.

The same GFLJ seedlings, as described above, were subjected to drought and salt stress. One-year-old healthy seedlings were chosen and acclimated to hydroponic culture with a 50% Hoagland [18] nutrient solution for two weeks, then switched to 100% for another two weeks in a constant-temperature light incubator under the following conditions: temperature (23 ± 2) °C, relative humidity 40–50%, light intensity 1000 μmol·m^−2^·s^−1^, and a 14 h·d^−1^ photoperiod. Seedlings with uniform growth underwent drought stress (25% PEG 6000) or salt stress (300 mmol·L^−1^ NaCl). Leaf samples were collected at 0, 3, 12, 24, 48, and 80 h after stress induction, with the 0 h sample as the control. For each treatment, leaves from 3 to 5 seedlings were pooled into one biological replicate. The mixed samples were quickly frozen in liquid nitrogen and stored at −80 °C, with three replicates for each time point.

### 2.2. Identification and Chromosomal Distribution of the LjDFR Gene Family

Genome sequences, protein sequences, and genome annotation files (Project ID: PRJCA001719) for *L*. *japonica* were downloaded from the National Genomics Data Center (https://bigd.big.ac.cn/gwh, accessed on 21 October 2025) [19]. *Arabidopsis thaliana* DFR protein sequences were obtained from TAIR (https://www.arabidopsis.org/, accessed on 21 October 2025). DFR protein sequences from *C. annuum*, *C. sinensis*, and *Brassica napus* were retrieved from the literature [16]. The AtDFR protein sequences were used for a BLASTP search against the *L*. *japonica* database. Sequences were screened based on the following criteria: >50% sequence homology, an E-value < 1 × 10^−50^, a bit-score > 300, and duplicates were removed. Two online databases—SMART (http://smart.emblheidelberg.de/, accessed on 22 October 2025) and CD-Search Tool (https://www.ncbi.nlm.nih.gov/Structure/bwrpsb/bwrpsb.cgi, accessed on 22 October 2025) were used for verification, excluding sequences without the complete DFR conserved domain. The chromosomal location of *LjDFR* genes was obtained from the genome annotation file (GFF) and visualized using TBtools-II v2.371 software.

### 2.3. Cloning, Physicochemical Properties, and Structural Characteristic Analysis of LjDFR Genes

Specific primers were designed with Primer Premier 5 software based on the obtained gene sequences (Table S1). PCR amplification was performed using KOD enzyme (Toyobo, Osaka, Japan) with cDNA from *L. japonica* petals as the template. The 20 μL reaction system included 10 μL of 2× PCR buffer for KOD FX, 4 μL of 2 mM dNTPs, 2 μL of cDNA template, 0.6 μL each of forward and reverse primers, 0.4 μL of KOD FX, and 2.4 μL of sterile double-distilled water. The reaction program was as follows: pre-denaturation at 94 °C for 2 min; 35 cycles of denaturation at 98 °C for 10 s, annealing at 55 °C for 30 s, and extension at 68 °C for 2 min; and a final extension at 68 °C for 5 min. PCR products were ligated into the T-vector according to the method described previously [20] and sent to Henan Youkang Biotechnology for sequencing. The resulting nucleotide sequences were imported into DNAMAN 6.0 to derive amino acid sequences. Physicochemical properties of LjDFR proteins, like amino acid count, molecular weight, theoretical isoelectric point and hydrophilicity/hydrophobicity were predicted using the Protein Parameter Calc tool in TBtools-II v2.371. Subcellular localization was predicted via Plant-mPLoc (http://www.csbio.sjtu.edu.cn/bioinf/plant-multi/, accessed on 23 October 2025). Secondary structure predictions used Prabi (https://npsa-prabi.ibcp.fr/cgi-bin/npsa_automat.pl?page=/NPSA/npsa_sopma.html, accessed on 23 October 2025). Tertiary structure models were constructed using SWISS-MODEL (https://swissmodel.expasy.org/interactive, accessed on 24 October 2025). Transmembrane domains were analyzed with TMHMM-2.0 (https://services.healthtech.dtu.dk/services/TMHMM-2.0/, accessed on 24 October 2025).

### 2.4. Subcellular Localization Analysis

The full-length CDS of *LjDFR3* and *LjDFR6* without stop codons were amplified using primers with homologous arms and adapters. They were inserted into pCAMBIA1300-35S-GFP by homologous recombination using the ClonExpress^®^ II One-Step Cloning Kit (Vazyme, Nanjing, China). The fusion vectors were transformed into the abaxial side of 3-week-old tobacco (*Nicotiana benthamiana*) leaves via *Agrobacterium tumefaciens* strain GV3101. The fluorescence signal with 488 excitation light was detected using a Zeiss LSM710 confocal laser scanning microscope (Zeiss, Oberkochen, Germany).

### 2.5. Multiple Sequence Alignment and Phylogenetic Analysis

Multiple sequence alignment of DFR proteins from *L*. *japonica*, *A. thaliana*, *C. annuum*, *C. sinensis*, and *B. napus* was performed using ClustalW in MEGA 7.0 software. A phylogenetic tree was built using the Neighbor-joining method with Bootstrap set to 1000. The tree was optimized using Evolview 3.0 online software and Adobe Illustrator 2020. Multiple sequence alignment of LjDFR proteins was conducted using DNAMAN6.0 and visualized with GeneDoc 2.7 and Adobe Illustrator 2020.

### 2.6. Conserved Motif and Gene Structure Analysis

Amino acid sequences of LjDFR proteins were submitted to the online tool MEME 5.5.2 (http://meme-suite.org/tools/meme, accessed on 24 October 2025) for conserved motif analysis. To prioritize the most statistically significant motifs and to facilitate a clear and interpretable downstream analysis, the maximum number of motifs was set to 10, with motif widths constrained to a range of 6–50 amino acids. The exon–intron structure of *LjDFR* genes was analyzed using GSDS 2.0 (http://gsds.gao-lab.org/, accessed on 24 October 2025), and results were visualized using TBtools-II v2.371 [21].

### 2.7. Collinearity Analysis and Identification of Cis-Acting Elements in Promoters of the LjDFR Gene Family

Collinear relationships among the *LjDFR* gene family in the *L*. *japonica* genome were analyzed using One Step MCScanX [22] in TBtools-II v2.371 and visualized via the Advance Circos module. The 2000 bp sequences upstream of the ATG start codon of *LjDFR* genes were extracted from the *L*. *japonica* database. *Cis*-acting elements were analyzed using PlantCARE (https://bioinformatics.psb.ugent.be/webtools/plantcare/html/, accessed on 24 October 2025), and the screened cis-acting regulatory elements were visualized using TBtools.

### 2.8. RNA Extraction, cDNA Synthesis, and qRT-PCR Analysis

Total RNA was extracted from roots, stems, leaves, flowers at various developmental stages of both *L*. *japonica* varieties and stress-treated samples using the FastPure^®^ Universal Plant Total RNA Isolation Kit (Vazyme, Nanjing, China). RNA quality and integrity were assessed by 1.1% agarose gel electrophoresis, and concentration/purity were measured with a NanoDrop 2000 spectrophotometer (Thermo Fisher, Waltham, MA, USA). Total RNA (1 μg) was used for reverse transcription to synthesize cDNA with the HiScript^®^ III 1st Strand cDNA Synthesis Kit (+gDNA wiper) (Vazyme, Nanjing, China). Fluorescent quantitative primers were designed with Primer Premier 5 based on conserved regions of the cDNA sequences (Table S1). The 10 μL qRT-PCR reaction system included 5 μL of RealStar Fast SYBR qPCR Mix (2×) (GenStar, Beijing, China), 1 μL of cDNA (10×), 0.3 μL each of forward and reverse primers (10 μmol·L^−1^), and 3.4 μL of RNase-free ddH_2_O. The reaction program was as follows: pre-denaturation at 95 °C for 2 min; 40 cycles of denaturation at 95 °C for 15 s and annealing/extension at 60 °C for 30 s. Amplification was performed on a QIAquant 96 2 plex real-time Detection System (Qiagen, Hilden, Germany), with melting curve analysis for specificity. Three biological and three technical replicates were used. Gene expression was normalized to the reference gene *LjG6PD* [23], which also served as a positive control. Additionally, no-template controls (NTCs) with nuclease-free water instead of cDNA were included in every run to check for contamination. The 2^−ΔΔCT^ method was used for quantification. The experimental data are shown as mean ± SD of three biological replicates. Tukey’s test was used for significance analysis (*p* < 0.05; *p* < 0.01).

## 3. Results

### 3.1. Genome-Wide Identification, Full-Length Cloning, and Chromosomal Distribution of the LjDFR Gene Family

**Figure 2:**
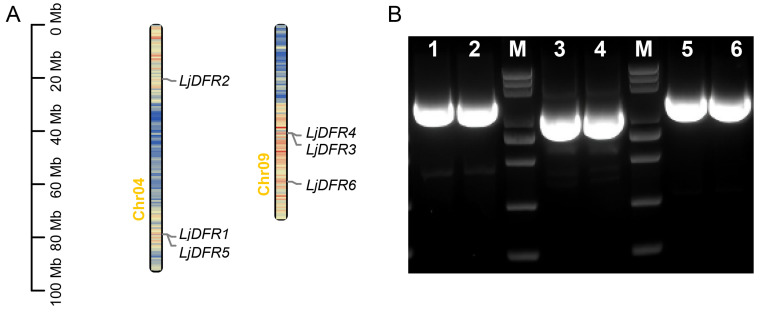
Distribution of *LjDFR* family members on chromosome map (**A**) and PCR amplification of *LjDFR* genes (**B**). 1–6: *LjDFR1*-*LjDFR6*; M: DL 5000 marker (5000, 3000, 2000, 1000, 750, 500, 250, 100 bp).

A total of six *DFR* genes were identified in *L*. *japonica*. Chromosomal localization analysis showed that these genes are distributed on Chr.04 and Chr.09 (Figure 2A). Specifically, *LjDFR1*, *LjDFR2*, and *LjDFR5* are on Chr.04, while *LjDFR3*, *LjDFR4*, and *LjDFR6* are on Chr.09.

To ensure the accuracy of later bioinformatics analysis, we cloned all six *LjDFR* genes (Figure 2B). The products were ligated into T-vectors for sequencing. Results showed 93.62% to 94.05% identity with the reference sequences. The 5′ and 3′ ends had high identity, but significant differences appeared in the middle sequences, including long-fragment insertions in some genes, likely due to varietal differences between the reference genome and the cloned material.

### 3.2. Protein Physicochemical Properties and Structural Analysis of the LjDFR Gene Family

The analysis of cloned LjDFR proteins showed variations in length, ranging from 328 (LjDFR3, LjDFR4) to 391 amino acids (LjDFR2), with an average of 350.5 amino acids (Table S2). Molecular weights ranged from 36.693 kDa (LjDFR4) to 43.686 kDa (LjDFR2), and the theoretical isoelectric point (pI) ranged from 5.82 (LjDFR2) to 8.28 (LjDFR5). All LjDFR proteins were hydrophilic, as indicated by negative GRAVY values. Subcellular localization prediction suggested that LjDFR1-LjDFR5 are in the cytoplasm, while LjDFR6 is nuclear (Table S2).

**Figure 3:**
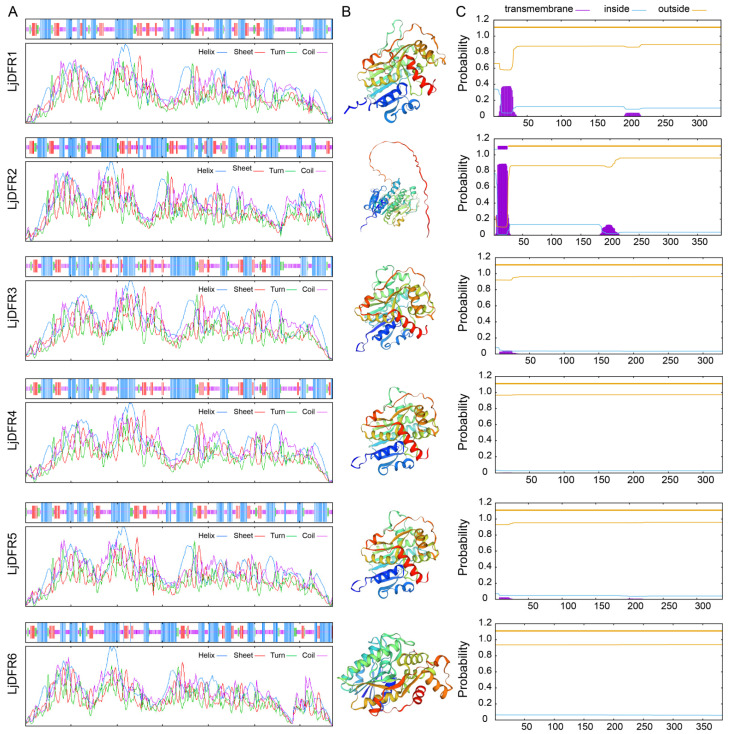
Prediction of secondary structure (**A**), tertiary structure (**B**) and transmembrane structure (**C**) of LjDFR proteins.

Secondary structure prediction revealed that all family members contain α-helix, β-turn, random coil, and extended strand. Except for LjDFR2, which has the highest random coil at 40.15%, all other members have the most α-helix ranging from 39.35% (LjDFR5) to 42.07% (LjDFR4). Additionally, β-sheets are the lowest among all members (Figure 3A). Tertiary structure results showed a significant presence of α-helix and random coil, consistent with the secondary structure predictions. The space between inversely parallel β-sheets can bind specifically to DNA sequences (Figure 3B). Transmembrane structure prediction revealed that only LjDFR2 has one transmembrane domain (1–6 aa), with the N-terminus intracellularly (7–24 aa) and the C-terminus extracellularly. Other LjDFR members lack transmembrane domains (Figure 3C).

### 3.3. Subcellular Localization Analysis of LjDFR Proteins

**Figure 4:**
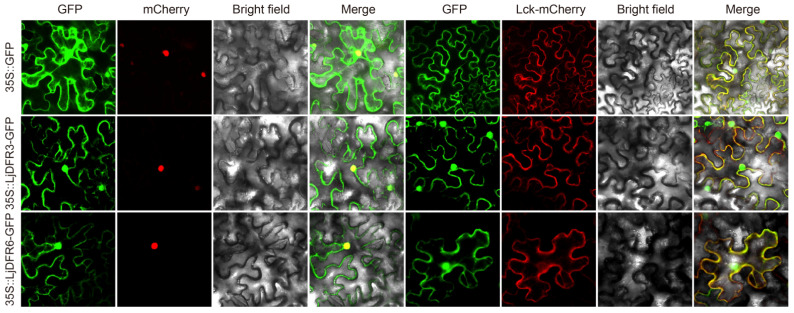
Subcellular localization of LjDFR proteins. GFP indicates the green fluorescence field, mCherry stands for the nuclear marker, Lck-mCherry stands for the membrane marker, Bright field indicates the bright field images, and Merge stands for the superimposed field. Scale bar = 20 μm.

To verify subcellular localization, we constructed recombinant 35S::LjDFR3-GFP and 35S::LjDFR6-GFP plasmids for transient expression in tobacco epidermal cells. The control (35S::GFP) showed diffuse fluorescence in the nuclear, cell membrane, and cytoplasm (Figure 4). In contrast, the fluorescence signals of 35S::LjDFR3-GFP and 35S::LjDFR6-GFP colocalized with cell membrane and nuclear marker fluorescence, confirming their localization.

### 3.4. Phylogenetic Analysis of the LjDFR Family

Multiple sequence alignment was performed to explore the evolutionary conservation of *LjDFR* genes. The N-terminal region of LjDFR proteins has a conserved NADPH-binding motif, while the C-terminal regions are more variable (Figure S1). Based on the residue at position 134, LjDFR2 is Asn-type, while the other five are non-Asn/non-Asp type (Figure S1).

**Figure 5:**
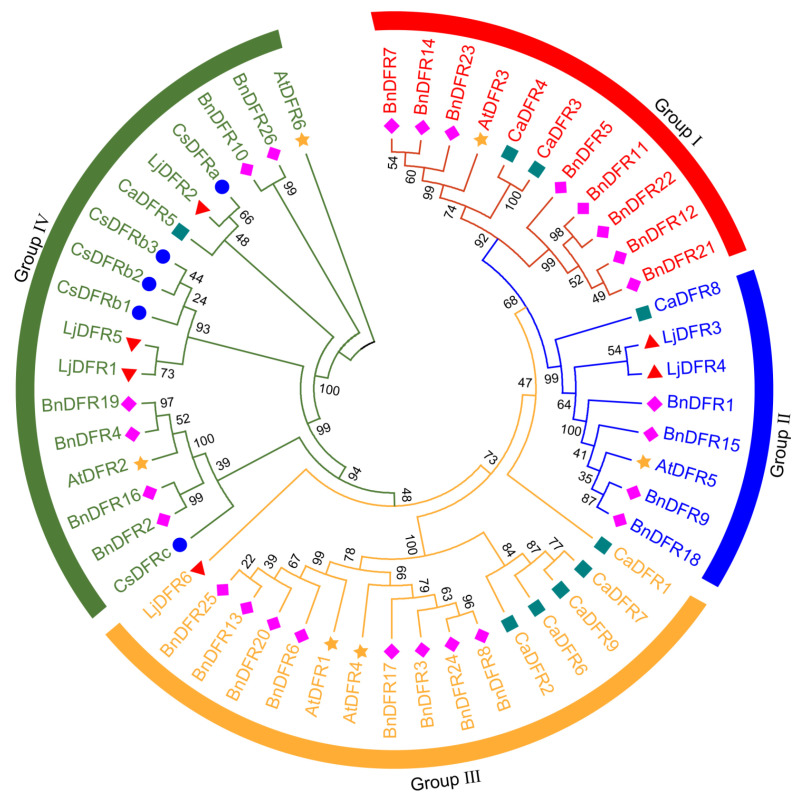
Phylogenetic tree of DFRs in *L*. *japonica* (Lj, ▲), *C*. *sinensis* (Cs, ●), *C*. *annuum* (Ca, ■), *B*. (Bn, ♦) and *A. thaliana* (At, ★).

Phylogenetic analysis of DFR proteins from four species grouped LjDFR proteins into four subfamilies (Groups I–IV), showing high conservation in plant evolution. Notably, no LjDFR proteins were found in Group I; two were in Group II, one in Group III, and three in Group IV (Figure 5).

### 3.5. Gene Structure, Conserved Motifs, and Synteny Analysis of the LjDFR Gene Family

**Figure 6:**
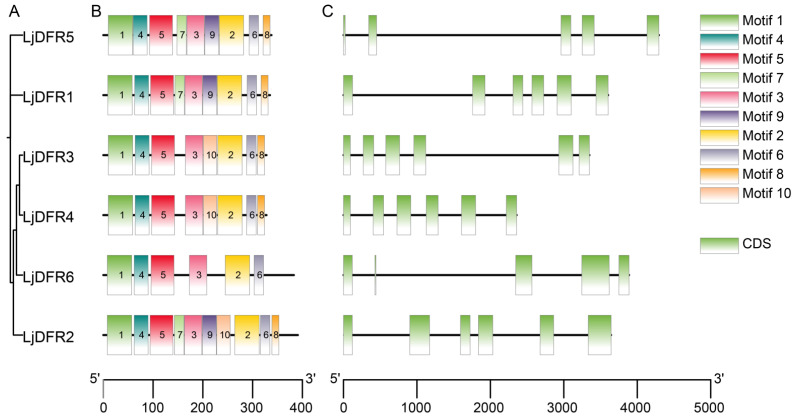
Phylogenetic tree (**A**), conserved motifs (**B**) and gene structure (**C**) analysis of the *LjDFR* gene family. (**A**) The phylogenetic tree of LjDFR proteins; (**B**) the conserved motifs of LjDFR proteins, Motif 1–10 in different colored blocks represent the motif composition; (**C**) gene structure of *LjDFR* genes. CDS: coding sequence.

We analyzed conserved motifs and gene structures alongside the phylogenetic tree (Figure 6A). Motif types and numbers were highly similar within the same subgroup but varied between different subgroups. LjDFR1 and LjDFR5 contained motifs 1–9, missing motif 10; LjDFR2 contained all 10 motifs; LjDFR3 and LjDFR4 lacked motif 7 and 9; and LjDFR6 had motifs 1–6 (Figure 6B). Gene structure analysis showed *LjDFR* genes have five to six exons (Figure 6C). *LjDFR5* and *LjDFR6* have five exons, while others have six. The similarities in gene structure within subgroups support the reliability of the evolutionary classification.

Gene duplication events are key in evolution. New genes from duplication can provide new functions or traits, aiding species’ development. Collinearity analysis helps reveal evolutionary relationships and gene duplication events. In this study, collinearity analysis showed no collinear relationships among the *LjDFR* genes (Figure S2). This suggests a lack of recent gene duplication events in this family in *L*. *japonica*, indicating a flexible evolutionary strategy.

### 3.6. Analysis of Cis-Acting Elements in the Promoters of LjDFR Genes

**Figure 7:**
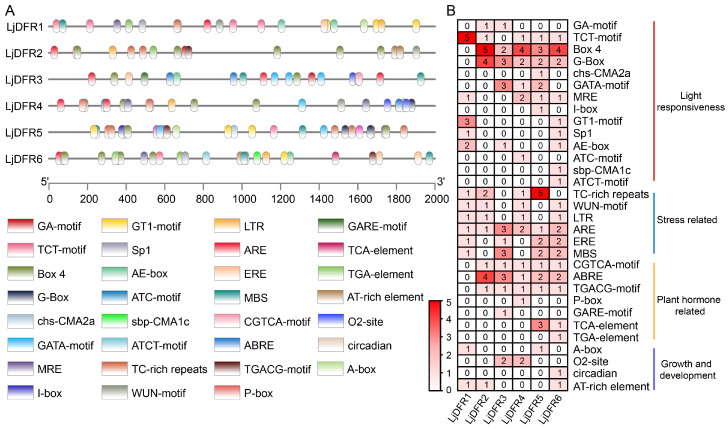
*Cis*-elements in the *LjDFR* gene promoters. (**A**) The location of the promoter *cis*-acting elements. (**B**) Statistical analysis of the number of *cis*-acting elements in the promoter region of the *LjDFR* gene family.

Analysis of the 2000 bp promoter regions upstream of *LjDFR* genes identified 148 *cis*-acting elements belonging to 31 types, grouped into four major categories (Figure 7). Light-responsive elements included 14 types, with Box4 being the most abundant (12.16%). Stress-responsive elements had six types, with the antioxidant response element ARE being the most numerous (6.76%). Hormone-responsive elements included seven types, with the abscisic acid-responsive element ABRE being the most abundant (8.11%). Elements related to plant growth and development included four types, with O_2_-site being the most numerous (2.70%). Notably, *LjDFR1* lacked hormone-responsive elements, while all other *LjDFR* promoters contained all four types. The composition of major elements varied among genes (e.g., *LjDFR1* had many TCT-motifs; *LjDFR2*, *LjDFR4*, *LjDFR6* rich in Box 4; *LjDFR3* rich in G-Box, GATA-motif, ABRE, ARE, MBS; *LjDFR5* rich in TC-rich repeats) (Figure 7). These results suggest *LjDFR* genes are involved in processes like photosynthesis, growth, development and responses to hormones and abiotic stresses.

### 3.7. Tissue Expression Patterns of the LjDFR Gene Family

**Figure 8:**
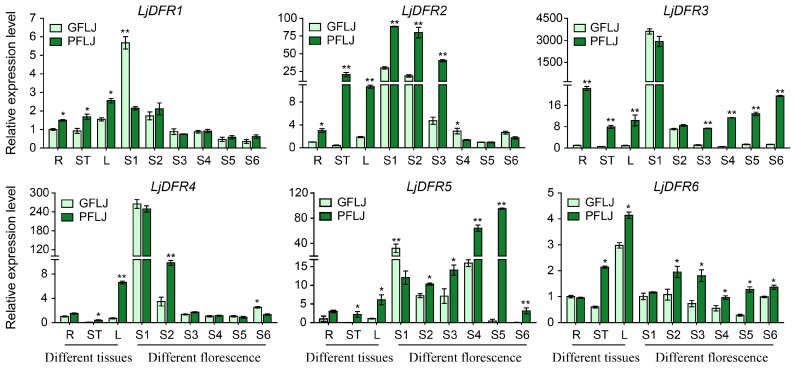
Differential expression patterns of *LjDFR* genes in different tissues and different florescence petals in GFLJ and RFLJ. All the data indicate means ± SD of three replicates. * indicates *p* < 0.05, ** indicates *p* < 0.01.

The qRT-PCR analysis revealed that *LjDFR* gene expression was tissue-specific in both GFLJ and RFLJ (Figure 8). Specifically, *LjDFR1* had the highest expression in the flowers of GFLJ and leaves of RFLJ. *LjDFR6* showed the highest expression in the leaves of both lines. *LjDFR2*, *LjDFR3*, and *LjDFR4* had the highest expression in the flowers of both lines. *LjDFR5* was highest in the stems of GFLJ and roots of RFLJ (Figure 8).

Furthermore, the expression trends of all *LjDFR* genes were consistent in a concordant manner across different flower developmental stages in both GFLJ and RFLJ. Notably, *LjDFR1* expression gradually decreased from S1 in both lines. *LjDFR2*, *LjDFR3*, and *LjDFR4* initially decreased, then increased, with the lowest levels at S5, S4/S3, and S5, respectively. *LjDFR5* showed a decrease–increase–decrease trend, lowest at S6, whereas *LjDFR6* exhibited an increase–decrease–increase pattern, with the lowest levels at S5 in GFLJ and S4 in RFLJ (Figure 8).

### 3.8. Expression Patterns of the LjDFR Gene Family Under Drought and Salt Stress

**Figure 9:**
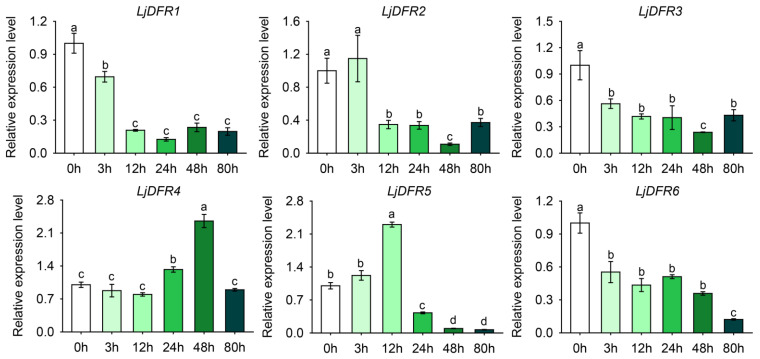
Expression patterns of *LjDFR* genes in *L*. *japonica* under drought treatment. All the data indicate means ± SD of three replicates. Different letters represented significant difference in purity (*p* < 0.05).

The expression patterns of *LjDFR* genes under drought and salt stress were analyzed to investigate the response of *LjDFR* genes to abiotic stress. Results showed differences in expression after stress exposure. Under drought stress, *LjDFR2*, *LjDFR4*, and *LjDFR5* were upregulated during treatment (Figure 9). *LjDFR2* expression showed an increase–decrease–increase trend. After 3 h of stress, expression rose sharply to 1.2 times the control, decreased thereafter and was significantly lower at 48 h. A slight upregulation occurred at 72 h. *LjDFR4* expression initially decreased, peaked at 48 h (2.4-fold), and then decreased. *LjDFR5* increased, peaking at 12 h (2.3-fold) before declining. In contrast, *LjDFR1*, *LjDFR3*, and *LjDFR6* were downregulated throughout the treatment period, and their relative expression trends were similar.

**Figure 10:**
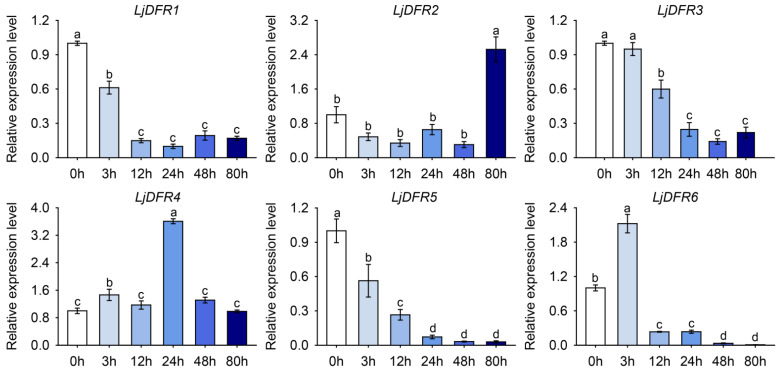
Expression patterns of *LjDFR* genes in *L*. *japonica* under NaCl treatment. All the data indicate means ± SD of three replicates. Different letters represented significant difference in purity (*p* < 0.05).

Under salt stress, *LjDFR2*, *LjDFR4*, and *LjDFR6* were upregulated (Figure 10). *LjDFR2* expression decreased initially and then increased, peaking at 72 h (2.5-fold). *LjDFR4* rose sharply at 3 h, then decreased, reaching a maximum at 24 h (3.6-fold). *LjDFR6* also increased sharply at 3 h (2.1-fold), followed by a gradual decline. Meanwhile, *LjDFR1*, *LjDFR3*, and *LjDFR5* showed downregulated expression to varying degrees during treatment (Figure 10).

## 4. Discussion

Flower color is vital in ornamental plants and a key breeding focus. It is mainly determined by the accumulation and combination of pigments like chlorophyll, carotenoids, and anthocyanins [2]. DFR is the first enzyme in anthocyanin biosynthesis and is essential for color. If DFR fails, anthocyanin production halts, directly affecting color. The first *DFR* gene was identified in maize and has been used in genetic engineering to alter flower color in petunias [24]. Overexpressing *HvDFR* in tobacco deepened its flower color [25]. Additionally, overexpressing *OjDFR1* in the *AtDFR* mutant tt3-1 restored anthocyanin levels and darkened flower color by upregulating *NtANS* and *NtUFGT* expression [26]. Transgenic *RdDFR1* also restored anthocyanin biosynthesis defects in seed coat, hypocotyls, and cotyledons of tt3-1 and changed tobacco flower color from pale pink to deep pink [27]. This study identified six *DFR* genes in *L*. *japonica*, comparable to *C. sinensis* (five members) [17] but fewer than *C. annuum* (nine members) [16].

Gene structure is crucial for gene family evolution. Structural differences contribute to divergence and new gene functions. The structure of *DFR* genes relates to their functions [28]. The amino acid sequences of LjDFR proteins range from 328 to 391 amino acids, averaging 350.5; this is similar to DFR sequences in many other plants [29]. Neutral amino acids outnumber acidic and basic ones, which might help form α-helix structures. All six LjDFR proteins have over 39% α-helix content. Except for LjDFR2, α-helix is the most common secondary structure in the other five proteins. Protein folding affects function by shaping characteristics and structure. All LjDFR proteins are hydrophilic, likely due to their high α-helix and random coil content [30]. Similar DFR structures are found in *Brassica oleracea*, *Glycine max*, and *Meconopsis* [31,32,33]. Most *LjDFR* genes have six exons, while two (*LjDFR5*, *LjDFR6*) have five, hinting at potential functional divergence.

Moreover, DFR function relates to its action site. Subcellular localization studies showed LjDFR3 and LjDFR6 in the cell membrane and nucleus, similar to DFRs in *Hosta ventricosa* [25] and *B. oleracea* [34], but different from cytoplasmic localization in *Loropetalum chinense* [35,36]. This indicates various localization patterns for DFRs across species. Anthocyanins synthesize mainly in the cytoplasm and move to vacuoles for storage via transport proteins. Thus, *DFR* genes may function in multiple organelles.

*LjDFR* genes showed tissue-specific and developmentally regulated expression. Most had the highest expression in flowers, then leaves, consistent with findings in *H. ventricosa* [25] and *Carthamus tinctorius* [29]. During flower development (S1–S6), most *LjDFR* genes showed decreased expression from S1, lowest at S5, then a slight rise at S6, similar to the findings in *Meconopsis* [33] and *Cineraria* [37], but inconsistent with *HvDFR*, which rises from S1 to S3, then decreases [25]. These results highlight that *DFR* gene expression is regulated differently by species and stages.

Notably, *LjDFR* expression levels were generally higher in RFLJ than in GFLJ, especially in pigmented tissues and specific developmental stages. All *LjDFR* genes in RFLJ stems and leaves were significantly higher than in GFLJ. The expression levels of *LjDFR2*, *LjDFR3*, *LjDFR4*, *LjDFR5* and *LjDFR6* in RFLJ were significantly higher at various stages compared to GFLJ, for instance *LjDFR2* expression in RFLJ was significantly higher at S1–S3 than in GFLJ; *LjDFR3* was higher at S3–S6; *LjDFR4* was higher at S2; *LjDFR5* and *LjDFR6* were significantly higher at S2–S6. This link between higher *DFR* expression and darker pigmentation aligns with observations in *Rhododendron hybridum* [38] and *Chrysanthemum morifolium* [39,40]. Phylogenetically, LjDFR2 aligns closely with functionally characterized DFRs CsDFRa [17] and CaDFR5 [16]. Previous studies confirmed that CsDFRa has DFR activity, converting dihydroflavonols into leucoanthocyanidins in vitro. Overexpressing *CsDFRa* in the *AtDFR* mutant (tt3) restored purple petiole color and improved seed coat color [17]. Additionally, *CaDFR5* expression levels matched anthocyanin content changes [16]. This, along with high expression in RFLJ, suggests that *LjDFR2* aids anthocyanin accumulation and flower color in *L*. *japonica*.

Promoters are DNA sequences upstream of a gene. They contain *cis*-acting elements that enhance or inhibit transcription and bind to transcription factors to regulate the gene expression under stress. Studying gene promoters is important for understanding plant gene functions. This study analyzed promoters and found many stress-responsive *cis* elements, leading to investigation under abiotic stress. Under drought stress, *LjDFR2*, *LjDFR4*, and *LjDFR5* showed increased expression during treatment, reaching 1.2-fold, 2.4-fold, and 2.3-fold of the control, respectively. Under salt stress, *LjDFR2*, *LjDFR4*, and *LjDFR6* also showed increased expression at 2.5-fold, 3.6-fold, and 2.1-fold of the control, respectively. Both *LjDFR2* and *LjDFR4* were significantly induced under both stresses, suggesting their potential roles in abiotic stress response. However, direct functional evidence is required to confirm this role. This lays the groundwork for further analyzing *LjDFR* gene functions.

## 5. Conclusions

This study identified six *LjDFR* genes in *L*. *japonica* through genome-wide analysis. Their full-length sequences were cloned, and analyses of chromosomal location, subcellular localization, protein structure, phylogenetic relationships, promoter *cis*-elements, and expression profiles were conducted. Results show that the *LjDFR* gene family is relatively conserved and involved in flower color formation and responses to drought and salt stress. Specifically, *LjDFR2* is key for anthocyanin accumulation, while *LjDFR2* and *LjDFR4* respond significantly to abiotic stress. This work provides a foundation for further characterization of *LjDFR* genes and offers candidate genes for molecular breeding aimed at improving ornamental traits and stress resistance in *L*. *japonica*. These findings are promising for plant physiologists and breeders, offering insights for enhancing drought and salinity tolerance in various crops.

## Data Availability

All data related to this study are open-access, and the databases, websites, and software information used have been detailed in the article and are available for interested researchers.

## Supplementary Materials

The following supporting information can be downloaded at: https://www.mdpi.com/article/10.3390/genes16121453/s1, Figure S1: Multiple sequence alignment of LjDFR proteins. The NADPH-binding site is boxed in green; Figure S2: Collinearity analysis of the *LjDFR* gene family; Table S1: Primers used in this study; Table S2: Physiochemical prosperities and subcellular location analysis results of LjDFRs.
