# Supplementary material for: Genome-Wide Identification of the DFR Gene Family in Lonicera japonica Thunb. and Response to Drought and Salt Stress
Source: Genes (Basel). 2025 Dec 4;16(12):1453. doi: 10.3390/genes16121453 (PMC12732910; doi:10.3390/genes16121453)
Supplement: Supplementary file 1 [file genes-16-01453-s001.zip › genes-4024586-supplementary.pdf]

Supporting Information

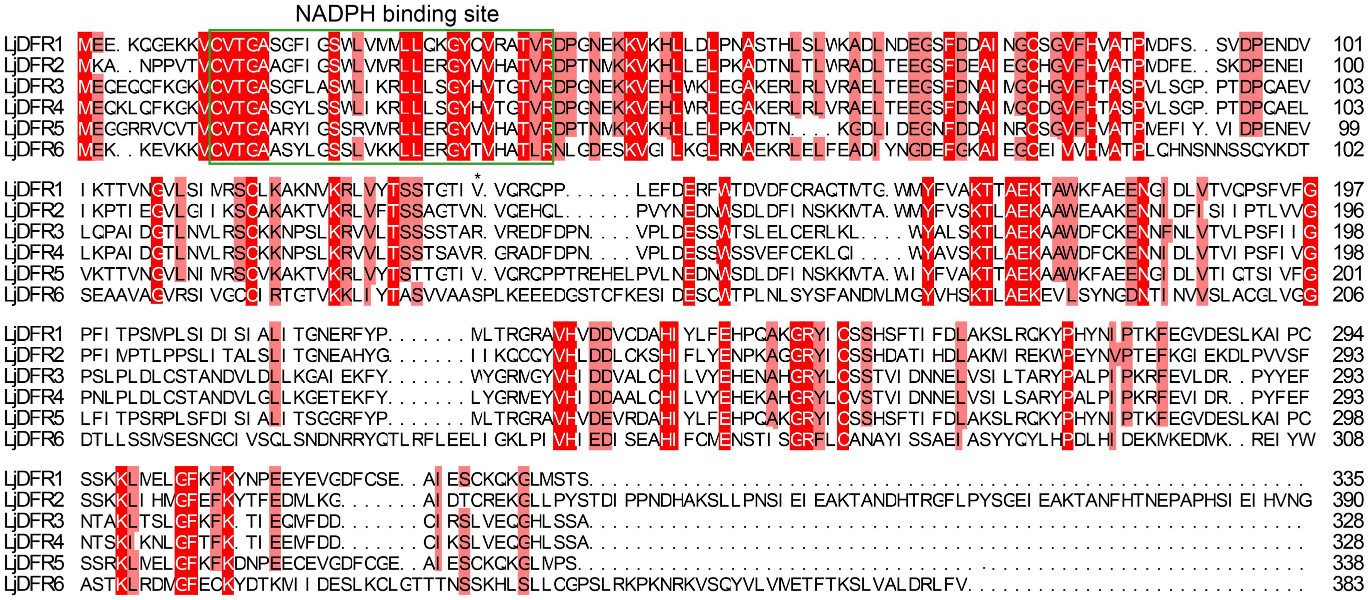

Figure S1. Multiple sequence alignment of LjDFR proteins. The NADPH-binding site is boxed in green.

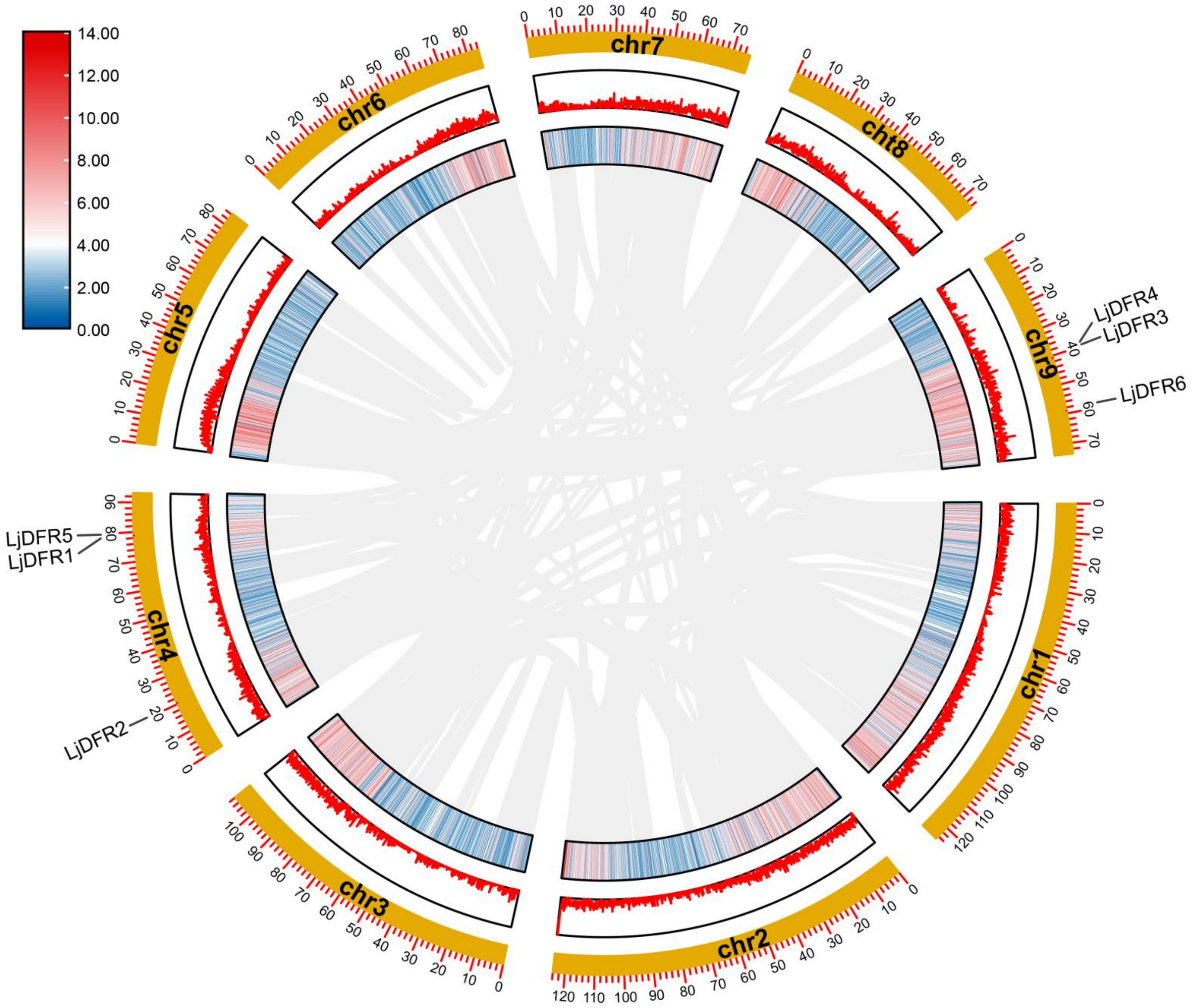

Figure S2. Collinearity analysis of the *LjDFR* gene family.

Table S1 Primers used in this study

Primer name	Primer sequence (5'-3')	Function
LjDFR1-F	ATGGAAGAAAAGCAAGGTGAAAAAAAAG	Gene amplification
LjDFR1-R	CTATGAAGTGGACATTAGCCCCTT	
LjDFR2-F	ATGAAAGCCAATCCCCCTGTC	
LjDFR2-R	TTACTCTGTGGGGACATTGTACTCG	
LjDFR3-F	ATGGAGCAAGAGCAACAATTCAAA	
LjDFR3-R	CTAGGCTGAAGAAAGATGGCCTT	
LjDFR4-F	ATGGAGCAAAAGCTGCAATT	
LjDFR4-R	CTAGGCTGAAGAGAGATGGCC	
LjDFR5-F	ATGGAAGGTGGAAGAAGGGTGTG	
LjDFR5-R	TTATGAAGGCATTAGCCCCTTTTG	
LjDFR6-F	ATGGAGAAAAAGGAGGTGAAGA	
LjDFR6-R	TTAGATGAATTCGTTCATCTTCTTG	
13GFP-LjDFR3-F	CAAATCGACTCTAGAAAGCTTATGGAGCAAGAGCAACAATTCAAA	Subcellular localization
13GFP-LjDFR3-R	GCCCTTGCTCACCATGGTACCGGCTGAAGAAAGATGGCCTT	
13GFP-LjDFR6-F	CAAATCGACTCTAGAAAGCTTATGGAGAAAAAGGAGGTGAAGA	
13GFP-LjDFR6-R	GCCCTTGCTCACCATGGTACCGATGAATTCGTTCATCTTCTTG	
LjG6PD-F	GACCCAACAGTTCCTGACAA	Reference gene
LjG6PD-R	GCTTTCCCTGCCTTGAGTATAA	
qLjDFR1-F	GTTACCGTTCAACCCTCTTTTG	Quantitative real-time PCR
qLjDFR1-R	CCTTAGCTTGTGGATGTTCAAAC	
qLjDFR2-F	CAGAAAGCAGCCTGGGAA	
qLjDFR2-R	TGATGAGTGAAAGAGCGGTAA	
qLjDFR3-F	GCTAGAAGGAGCAAAGGAGA	
qLjDFR3-R	GGGGAAGCAGTGTGAAAAA	
qLjDFR4-F	TGAGGAAGGTAGCTTTGACAAT	
qLjDFR4-R	CTTACGGCAGAAGTAGATGAGG	
qLjDFR5-F	AAGCAATCCCTTGTTCATCC	
qLjDFR5-R	CCCAACTTCACATTCCTCAG	
qLjDFR6-F	TGCTTGTGGTCTTGTGGG	
qLjDFR6-R	TGTCATTTGAGAGTTGCGAT	

Table S2 Physiochemical prosperities and subcellular location analysis results of LjDFRs

Gene name	Gene ID	Protein length/AA	Molecular Weight/Da	Theoretical pI	Instability Index	Aliphatic Index	Grand Average of Hydropathicity	Subcellular location
LjDFR1	Lj4C788T15.1	335	37619.16	6.26	30.42	75.91	-0.209	Cytoplasmic
LjDFR2	Lj4A204T70.1	391	43686.09	5.82	34.35	86.06	-0.22	Cytoplasmic
LjDFR3	Lj9A408T82.1	328	36830.22	6.02	38.82	93.02	-0.156	Cytoplasmic
LjDFR4	Lj9A407T55.1	328	36693.12	5.96	33.8	93.29	-0.15	Cytoplasmic
LjDFR5	Lj4C788G14.1	338	38041.83	8.28	30.65	83.58	-0.207	Cytoplasmic
LjDFR6	Lj9A590G56.1	383	42586.81	6.18	37	89.84	-0.188	Nuclear
